# Working memory capacity as a predictor of text idea recall in multitasking L2 digital reading

**DOI:** 10.1186/s41155-025-00353-2

**Published:** 2025-05-07

**Authors:** Bruno de Azevedo, Davi Alves Oliveira, Ingrid Finger, Leda Maria Braga Tomitch

**Affiliations:** 1 Department of Teaching, Research and Extension, Federal Institute of Santa Catarina - IFSC, Rua José Lino Kretzer, 608, Praia Comprida, 88103-902 São José, Santa Catarina Brazil; 2 Department of Human Sciences–Campus IV, State University of Bahia–UNEB, Rua J.J. Seabra, 158, Estação, 44700-000, Jacobina, Bahia Brazil; 3 Department of Modern Languages - UFRGS, National Council for Scientific and Technological Development - CNPq, Federal University of Rio Grande do Sul - UFRGS, Avenida Bento Gonçalves, 9500, Agronomia, 91540-000 Porto Alegre, Rio Grande Do Sul Brazil; 4 Graduate Program in English: Linguistic and Literary Studies, Federal University of Santa Catarina–UFSC, Campus Universitário Reitor João David Ferreira Lima, Florianópolis, Santa Catarina 88035-972 Brazil

**Keywords:** Bilingualism, Working memory capacity, Second language digital reading, Multitasking

## Abstract

**Background:**

Digital reading in a second language is a cognitively demanding task constrained, among other factors, by individuals’ working memory resources. Additionally, listening to music whilst reading might overload working memory. However, previous studies found mixed results regarding the effects of music in reading comprehension.

**Objective:**

This study investigates the effects of working memory capacity on English as a second language digital reading comprehension in a multitasking setting. More specifically, it examines how working memory capacity influences the ability to recall text ideas (main ideas, secondary ideas, and details) after reading a digital text in a second language and simultaneously listening to music.

**Method:**

The between-subject design study was conducted with sixty-five participants aged 19–62 years (*M* = 28.87; SD = 8.20) divided into two groups of Brazilian Portuguese-English proficient bilinguals, one reading while listening to lyrical music and the other reading while listening to non-lyrical music. Participants’ working memory capacity was assessed using a Self-Applicable version of the Reading Span Test, and their reading comprehension of a second language digital text was assessed with a free-written recall. Participants also rated their proficiency in their L2.

**Results:**

Findings suggest that individuals with higher working memory capacity are better at recalling secondary ideas and details, while no effect was observed in the recall of main ideas. Additionally, no difference between groups (lyrical vs non-lyrical) was observed. An exploratory complementary analysis indicates that readers who recall secondary ideas tend to better recall main ideas, so higher working memory capacity seems to enable the recall of secondary ideas in addition to main ideas.

**Conclusion:**

This study contributes to understanding cognitive resource allocation in second language digital reading and the potential effects of multitasking on reading comprehension.

## Introduction

Making sense of everyday input demands processing in working memory (Logie et al., 2021), the system held responsible for processing and maintaining information temporarily for carrying out complex cognitive tasks (Baddeley et al., 2021; Cowan et al., 2021). In addition, processing demands in working memory (WM) are prone to increasing when performing two complex tasks simultaneously (Azevedo et al., 2022; Baddeley et al., 2021). A clear-cut example of a complex cognitive task is comprehending a text, given that readers must manipulate an array of information in WM, involving both processing and storage, in order to construct a coherent mental representation of the text being read (van den Broek & Kendeou, 2022). More specifically, readers must keep active the theme of the text, representation of the text situation, and the preceded ideas from the text whilst making sense of the current idea from the text (Just & Carpenter, 1992). Such complexity is increased when reading in a second language (L2) in a digital format (DeStefano & LeFevre, 2007; Salmerón et al., 2018).

L2 reading is believed to be more cognitively demanding compared to L1 reading by monolinguals because it involves joint activation of both dominant and non-dominant language systems (Bialystok et al., 2012). For bilinguals, even though the L2 may also be activated during L1 reading (Kroll et al., 2021), the L1 tends to be the dominant language, and overall, bilinguals are typically more proficient in L1 than in L2. Consequently, a lower degree of automaticity may cause L2 reading to be more cognitively demanding than L1 reading, even for bilinguals. L2 proficiency interacts with WMC, in the sense that more proficient L2 users lessen the WM burden because they process linguistic and propositional aspects of a text efficiently and automatically, with minimal strain on their working memory (Alptekin & Erçetin, 2010, p. 214). Given WM’s limited capacity, a relationship between WM capacity in L2 reading comprehension is expected and has been observed in various studies (Linck et al., 2014). Reading in a digital environment is expected to further increase cognitive demands, as it requires navigating multiple sources, evaluating their reliability, and integrating information to construct a coherent representation (DeStefano & LeFevre, 2007; Wu, 2020). Thus, L2 digital reading, by bilinguals, can be considered more complex than L1 print reading by monolinguals.

In addition, in contexts in which there are multitasking demands, such complexity can increase even further, given attentional bottlenecks. Attention must be selective whilst performing simultaneous tasks resulting in bottlenecks that might lead to impairment in one or both tasks (Tombu et al., 2011). One example is reading while listening to music, as attention may shift between the text and the song. Put simply, cognitive resources are limited and this scenario might be aggravated when imposing several tasks for processing in the arena of computation—working memory (Just & Carpenter, 1992).

Given such processing complexity, a derived hypothesis is that individual differences in working memory capacity (WMC) could potentially serve as predictors of multitasking ability (Clinton‐Lisell, 2021; Pollard & Courage, 2017). In this study, we investigate multitasking by considering L2 reading in a digital format and listening to music simultaneously. Despite extensive research on the matter, results remain inconclusive. While some studies have found music not to be distracting for reading comprehension (Chitwood & Vaughn, 2018; Kou et al., 2018), others have suggested that music may hinder comprehension (Avila et al., 2012; Perham & Currie, 2014). Interestingly, a greater distracting effect of lyrical music as compared to non-lyrical music has also been found (Vasilev et al., 2023). Therefore, we added types of music as a variable in the investigation. Additionally, possible effects of WMC in the context of L2 digital reading, where language proficiency also plays a crucial role, are still open for debate.

### Review of literature

It is undeniable that there has been a shift in reading behaviors over the past decades which led researchers to theorize about the implications of digital reading on cognitive resources (DeStefano & LeFevre, 2007; Salmerón et al., 2018). One of the arguments is that cognitive resources might be overused to cope with digital reading, since readers must decide which links to click in addition to integrating several pieces of information from these multiple sources (DeStefano & LeFevre, 2007). Within cognitive resources, this study specifically focuses on WM, the short-lived cognitive capacity to hold and manipulate information necessary in complex cognitive processing (Baddeley et al., 2021; Cowan et al., 2021; Wen & Schwieter, 2022). Despite L2 reading differing from L1 in terms of linguistic and pragmatic knowledge, language exposure, among other factors, WM differs in processing L2 reading in comparison to L1 (Linck et al., 2014; In’nami, 2022). More specifically, working memory (WM) differs in processing L2 reading compared to L1 because performing WM tasks in a second language (L2) often adds extra cognitive demands that are not present when using the first language (L1). This is because the L1 is typically the dominant language, resulting in higher proficiency than the L2. In reading, not only must individuals store and process information but also manage language comprehension, which can be more difficult due to lower automaticity and proficiency in the L2. This increased effort means that WM tasks in L2 often reflect both memory capacity and language ability, making their relationship with L2 reading appear stronger. In contrast, WM tasks in L1 better isolate pure memory skills, as language comprehension is less of a barrier. Therefore, the language in which WM is tested plays a key role in how it relates to reading performance in L2 versus L1 (Prebianca, 2010).

Similarly, digital reading requires more advanced reading skills in comparison to traditional single-page linear reading, once readers must be able to (1) *navigate* across pages in order to select the suitable information sources in order to construct a coherent mental representation of what was read, (2) *integrate* information from multiple sources, and (3) *evaluate* the quality of information found (Salmerón et al., 2018). More specifically, efficient *navigation* demands more resources in visuospatial WM, which requires devoting additional time to thoroughly explore and compare the contents within the hypermedia environment from multiple perspectives (Salmerón et al., 2018). *Integration* is also compromised, especially for readers with lower previous knowledge and lower WM span (DeStefano & LeFevre, 2007).

In this regard, digital reading might be itself deemed as multitasking, once it “requires switching between different information sources using search engines to select, evaluate and integrate information across various web pages and media for complex information processing and decision making” (Wu, 2020, p. 876). Thus, it is hypothesized that multitasking might burden WM resources at a larger extent in comparison to reading printed materials, since readers must be able to navigate, integrate, and evaluate pieces of information from the digital environment at the same time they have to store and manipulate an array of information for constructing the mental representation of the text read. Besides, such text representation might be compromised when an additional task is added, such as listening to music whilst reading a text in an L2. To demonstrate, Johansson et al. (2012) investigated how different sound conditions could potentially affect onscreen reading in a group of monolingual university students whose native language was Swedish. Results showed that non-preferred music negatively influenced reading, as compared to silence, while liked-music and noise had no differences. WMC neither correlated with the eye-movement data nor with the reading comprehension scores, due to participants’ higher WMC, as the authors concluded.

Similarly, Vasilev et al. (2018) showed the detrimental effects of noise, speech, and music on reading comprehension in a Bayesian meta-analysis carried out with 65 studies. Interestingly, lyrical music was found to be more distracting as compared to non-lyrical music, in the same way that intelligible speech was more distracting than unintelligible speech. These findings are consistent with both the *semantic-interference hypothesis* (Martin et al., 1988) and the *interference-by-process hypothesis* (Marsh et al., 2009). The former posits that the semantic properties of background speech interfere with the reading material (Martin et al., 1988), while the latter argues that speech and reading compete for cognitive processing (Marsh et al., 2009). Thus, if lyrics are processed akin to speech, lyrical music should be similarly distracting (Vasilev et al., 2023).

### Current study

In this study, we investigated how Brazilian Portuguese-English adult bilinguals with different levels of WMC performed in a hypertext L2 reading task while simultaneously listening to music. We specifically focused on L2 digital reading comprehension, considering the additional burden imposed on the WM resources of bilinguals when individuals read in their L2 in a digital format. Comprehension was operationalized by the number of ideas recalled. In other words, the ability to recall ideas from the text after reading, without access to the text. Participants of this study self-rated their age of onset (age they started learning English), and age of fluency (when they became fluent in English) (*M*_onset_ = 10.3 and *M*_fluency_ = 18.9, respectively). Hence, in this study we refer to participants as bilinguals, but consider English their second language (L2), as their English acquisition occurred later in life (Ortega, 2013) and may be characterized as successive language acquisition, which can happen at any point in life (Li, 2013). Participants were divided into two groups, one in which they listened to lyrical music and the other to non-lyrical music. Both the text and the music stimuli were presented in their less dominant language—English. We hypothesized that higher WMC participants would outperform their lower WMC counterparts at recalling main ideas, secondary ideas, and details regardless of group. In addition, we also hypothesized that non-lyrical music (binaural beats, in this study) would be found to be less distracting compared to lyrical music. This assumption considers that binaural beats feature steady intensity (Garcia-Argibay et al., 2019) and that would cause less distraction (Vasilev et al., 2018), whereas lyrical music contains spoken words and convey semantic information, as predicted by the *semantic-interference hypothesis* and the i*nterference-by-process hypothesis*.

## Method

### Participants

Sixty-five Brazilian Portuguese-English bilingual adults aged 19–62 years (*M* = 28.87; SD = 8.20) participated in this study. It was conducted in a between-subject design that consisted of two groups: one exposed to lyrical music and the other to non-lyrical music. Gender and age distributions were balanced between the lyrical group (24 females, 7 males, *M*_age_ = 28.5, SD_age_ = 7.21) and the non-lyrical group (26 females, 8 males, *M*_age_ = 29.4, SD_age_ = 9.13), with no statistically significant difference observed in age between groups (*t*(61.77) = − 0.41, *p* = 0.682, 95% CI = [− 4.90, 3.22]). Invitations were sent out via a social networking website and email to university students from the undergraduate and graduate English Departments from Universidade Federal de Santa Catarina and English undergraduate students from Universidade Federal do Rio Grande do Sul. All procedures were approved (Protocol 4.688.798) by the Ethics Review Board of Universidade Federal de Santa Catarina (UFSC) in accordance with Resolutions 466/12 and 510/16 of the Brazilian National Council of Health. Consent procedures followed specific guidelines for experimental studies conducted online. The age of onset for L2 acquisition did not significantly differ (*t*(62.48) = − 0.27, *p* = 0.788, 95% CI = [− 2.49, 1.90]) between the lyrical group (*M*_onset_ = 10.44; SE_onset_ = 0.83) and non-lyrical group (*M*_onset_ = 10.15; SE_onset_ = 0.72). Similarly, no significant difference was observed in the age of onset fluency (*t*(62.81) = − 1.75, *p* = 0.085, 95% CI = [− 4.74, 0.32]) between the lyrical group (*M*_fluency_ = 19.97; SE_fluency_ = 0.94) and the non-lyrical group (*M*_fluency_ = 17.76, SE_fluency_ = 0.85). The pool consisted of proficient bilinguals whose native language is Brazilian Portuguese and for whom English is a second language, confirmed by the Language Experience and Proficiency Questionnaire (Scholl et al., 2021). In this questionnaire, participants’ self-reported English proficiency in reading, listening, speaking, and writing. No participant was excluded from the sample since they all met the criteria of being a proficient bilingual. As Table 1 shows, no significant differences were observed between participants’ self-reported English proficiency and they were all considered very proficient in English, where a score of 6 is deemed proficient, according to the self-reported proficiency scale used in this study, available at Open Science Framework (Azevedo & Oliveira, 2024).

**Table 1:** Self-reported proficiency

Skill	Lyrical		Non-lyrical	
	Median	IQR	Median	IQR
Reading	6	1	6	1
Listening	6	1	5	1
Speaking	5.5	1	5	2
Writing	5	1	5	2

Elaborated by the authors. A Kruskal–Wallis test indicated no significant difference in self-reported proficiency between groups, *χ*^2^ (1, *N* = 65) = 2.00, *p* = 0.158

### Materials and procedures

Data was collected remotely during the COVID-19 pandemic, thus the experiment was set up at Lapsi*—Laboratório de Psicolinguística na Web *(http://lapsi2.davi.solutions/ JavaScript platform which is also a repository of digital instruments for researchers in Psycholinguistics, Applied Linguistics and related areas, allowing data to be collected remotely. After consent, participants were briefed via tutorial, either in a written format or video, on how to perform the experimental tasks. As the experiment was conducted online, participants were informed that once initiated, it had to be completed in a single session; otherwise, their responses would not be saved. The link to access the online experiment was distributed via email.

WMC was measured by the Self-Applicable Reading Span Test (Oliveira et al., 2021) while reading performance was measured by the recall of text ideas. Text ideas were categorized into three levels: (1) main ideas, considered the ideas that were part of the thesis statement of the text; (2) secondary ideas, considered those that were used to support or evidence the main ideas; and (3) details, considered those ideas that could be removed from the text without altering its main ideas. We tested the hypotheses that readers with higher WMC should be better than readers with lower WMC at recalling main ideas, secondary ideas, and details from a text when reading in a multitasking condition. This condition was specifically L2 digital reading whilst listening to lyrical and non-lyrical music. Since lyrical music is expected to be more distracting than non-lyrical music (Vasilev et al., 2018), we expected an interaction between WMC and Group (lyrical vs non-lyrical) with WMC being a better predictor of recall in the lyrical group in comparison with the non-lyrical group.

Readers’ WMC was assessed using a variation of the widely known Reading Span Test (Daneman & Carpenter, 1980), the Self-Administrable version of the Reading Span Test (Oliveira et al., 2021) set up in the same platform the experiment was hosted (Lapsi). This version allows participants to self administer the test without the researchers’ intervention. The original test consists of participants reading 60 sentences out loud and trying to recall the last word of each sentence in the order they were originally presented. WMC is thus determined by the level at which the participant fails to recall the last word and/or by the total number of recalled words. In the Self-Administrable version of the Reading Span Test, participants must read the 60 sentences, select the word that best completes the sentence, and recall the selected word. The Self-Administrable version of the test randomly samples 60 sentences, from a selection of 180 sentences from a corpus composed of news, ranging from 9 to 15 words displayed in five different levels with increasing sets. Level 1 comprises two sentences per set, while level 5 encompasses six sentences within the set. Each level consists of three trials with the same number of sentences. To guarantee participants did not simply memorize each end-word, two buttons containing two end-words were added so that participants selected the word that best completes each sentence. This version did not require sentence reading aloud. The test was administered in Brazilian Portuguese to avoid L2 proficiency confounding the results (Linck et al., 2014; Tomitch, 2020).

The Self-Administrable version of the Reading Span Test was scored using two methods. The first, known as the lenient scoring method, disregards word order and the final score is the total number of correctly recorded words. The second, the strict scoring method, takes word order into account. The final score for this method is determined by the level at which the participant first failed to correctly recall at least two trials. If the participant managed to correctly recall one trial, an additional half point is added to their score. Despite the fact that the second method is widely used, the first method has been shown to have more normally distributed data, potentially yielding more robust statistical analyses (Friedman & Miyake, 2005). Therefore, a comparison of results using both methods can provide further insight into which method is more suitable in different contexts.

Idea recall was measured by a free written recall task. After reading a text, both in the lyrical and non-lyrical group, participants were asked to type in the corresponding box everything they could recall from the text. The text read was split by the first author into 57 idea units (IUs) that were classified into main ideas, secondary ideas, and details and then assessed by five independent raters. The raters received a booklet containing (1) a framework for rating the IUs; (2) the original text used in the experiment and (3) the text split into IUs. The framework was based on Tomitch (2012) and Kintsch (1998) and is summarized in Table 2. Interrater reliability was assessed using Fleiss’ kappa (Fleiss, 1971; Hallgren, 2012) and initially resulted in *k* = 0.13 (*p* < 0.001), indicating slight agreement (Landis & Koch, 1977). We identified that raters strongly disagreed regarding six IUs. Thus, an online meeting with the raters was scheduled to discuss these disagreements. Each of the six contentious IUs was presented in the context of their paragraph, and the raters were asked to privately select from the most popular options previously voted on. After this, the result from inter-rater reliability was *k* = 0.27 (*p* < 0.001), indicating fair agreement (Landis & Koch, 1977). Then, the category selected for each IU was the one indicated by at least half of the raters (rounded up). Ten of the IUs were classified as main ideas, thirty as secondary ideas, and seventeen as details. Correct recall of IUs was considered either the literal recall of the IU or a paraphrase of the IU (Tomitch, 2003).

**Table 2:** Framework for rating the idea units

Type	Questions
Main idea	What is the thesis statement? What is the controlling idea in the text? What is the main point the writer is trying to make?
Secondary ideas	What are the supporting arguments for the main point? What evidence is presented to support the main point?
Details	Is this idea relevant for the main idea or is a minor detail? (MI should not be affected by deletion of IUs containing details)

Elaborated by the authors, based on Kintsch (1998) and Tomitch (2012)

The reading stimuli consisted of an expository text retrieved from a journal that shares effective practices in language teaching and learning, available at http://www.ufrgs.br/revistabemlegal. To ensure consistency in text length, only one out of the four sections of the text was used, the one titled “English as a Lingua Franca: Implications for Language Policies and Pedagogical Practices”. The text was displayed in Times New Roman, Font size 12pt, to enhance readability (Darroch et al., 2005). As data was collected remotely, screen size may have differed across participants.

Readability was also controlled using the Flesch-Kincaid metrics and Text Ease and Readability Assessor, both provided by Coh-Metrix (http://cohmetrix.com/) (Graesser et al., 2004, 2011, 2014). Initial inspections showed a difficult text to read, especially in terms of syntactic complexity. The text was then manipulated to increase readability (Azevedo & Oliveira, 2024).

Participants were randomly assigned into the two groups: the lyrical group, who listened to songs with lyrics and the non-lyrical group, who listened to binaural beats. In both conditions, participants were able to listen to a 30-s excerpt of each of two available options per group. This setup was intended to mimic the real-life task of selecting a song before beginning to read. The lyrical group had to choose either Dua Lipa’s “Levitating” (103 *bpms*) or The Weeknd’s “Save your tears” (117 bpms), extracted from Spotify’s *Today’s Top Hits.* The non-lyrical group had to select either ‘Alpha Thoughts 107 Hz–114 Hz’ (101 bpms) or “Binaural Alpha Sinus” (120 bpms) from Spotify’s *Binaural Beats: Focus*. The tempo was adjusted using the *MixMeister BPM Analyzer* software, ranging from 108 to 120 bpms, which falls within the moderato tempo range (Fernández-Sotos et al., 2016), which do not seem to impair comprehension (Thompson et al., 2012). Participants were instructed to use earphones and listen to the song at a comfortable volume. They were also instructed to read the text within around 15 to 20 min to understand the main idea and as much information as possible. Materials can be accessed on the Open Science Framework (Azevedo & Oliveira, 2024).

Data was analyzed using the R language and statistical environment (R Core Team, 2023), using the *lm* function, with recall as the response variable and WMC, Group (lyrical and non-lyrical), and their interaction as predictors. The models varied depending on the category of IU considered: the first model considered the recall of main ideas, the second model considered the recall of secondary ideas and the third model considered the recall of details. Additionally, two versions of each model were analyzed: one using the strict scores of the WMC test, and the other using the lenient scores. For all tests, we use a significance level of 0.05.

## Results

We hypothesized that WMC would predict participants’ recall of main ideas, secondary ideas, and details of a hypertext in both groups, the lyrical music and the non-lyrical music group, being a better predictor in the lyrical group in comparison with the non-lyrical group.

**Fig. 1:**
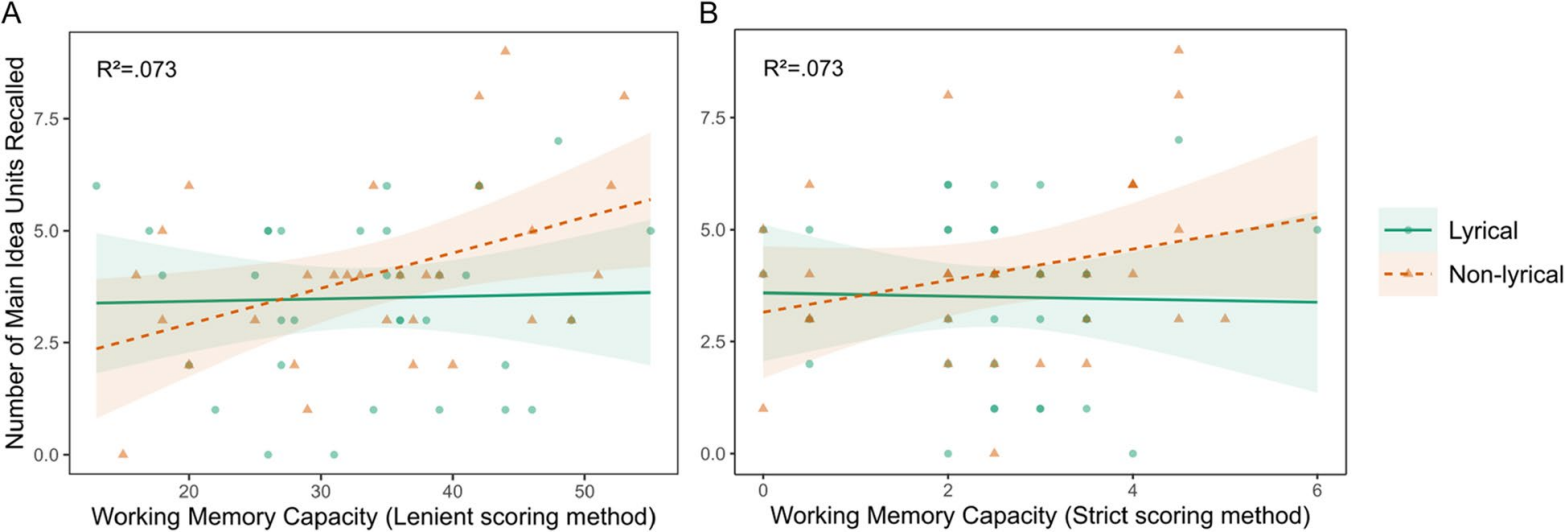
Recall of main ideas predicted by WMC. *Note.* WMC vs number of main idea units recalled, using the lenient method (**A**) and the strict method (**B**) for scoring the reading span test. Colors, shapes and line types differentiate between lyrical and non-lyrical groups. No statistically significant effect was observed

**Fig. 2:**
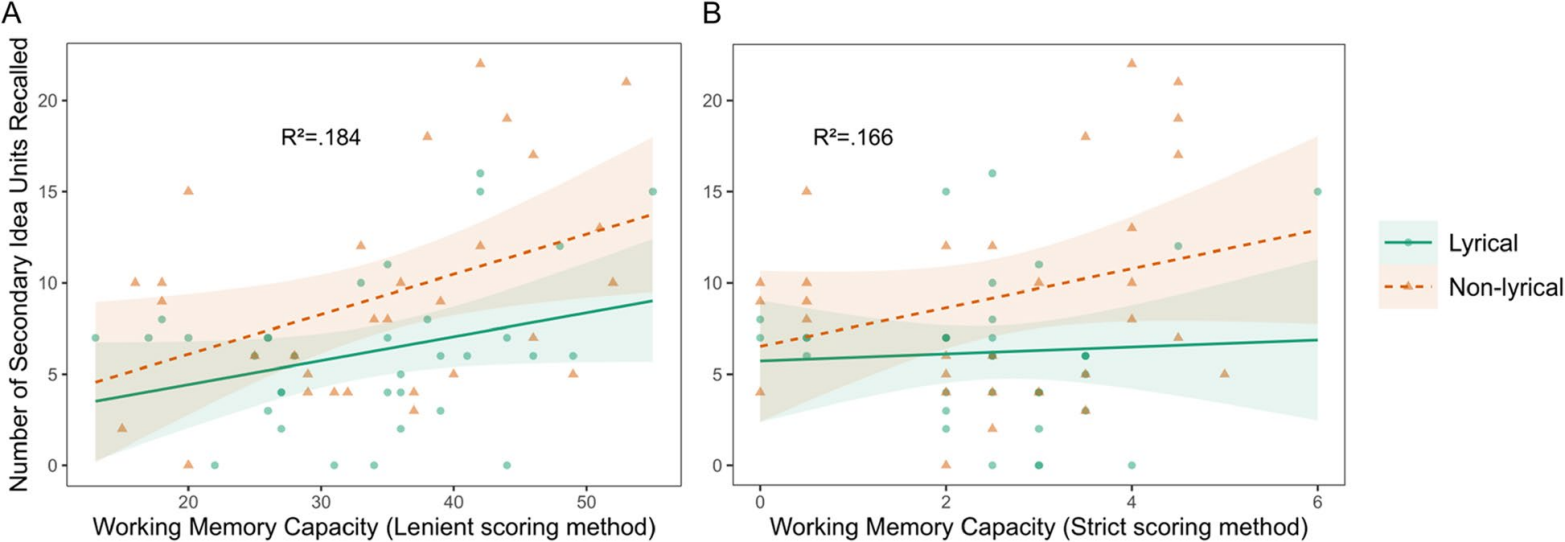
Recall of secondary ideas predicted by WMC. *Note.* WMC vs number of secondary idea units recalled, using the lenient method (**A**) and the strict method (**B**) for scoring the reading span test. Colors, shapes and line types differentiate between lyrical and non-lyrical groups. The effect of WMC was statistically significant with both scoring methods

No statistically significant effects were observed on predicting the recall of main ideas, neither using the lenient scoring method, nor the strict scoring method (Table 3, Fig. 1). However, a statistically significant effect of WMC in secondary ideas recall was obtained both with the lenient scoring method and the strict scoring method (Table 4, Fig. 2).

**Table 3:** Estimates of working memory capacity and group as predictors of main idea recall

Variable	Lenient method			Strict method		
	*β*	*p*	95% CI	*β*	*p*	95% CI
Intercept	1.10	0.53	[− 2.4, 4.6]	2.30	0.03	[0.23, 4.4]
WMC	0.06	0.09	[− 0.02, 0.14]	0.48	0.08	[− 0.05, 1]
Group	1.20	0.62	[− 3.5, 5.9]	1.60	0.24	[− 1.1, 4.2]
Interaction	−0.04	0.48	[− 0.14, 0.07]	− 0.60	0.10	[− 1.3, 0.11]

Formula: “Main idea recall” ~ WMC * Group

^*^Statistically significant effect (*p* <.05)

**Table 4:** Estimates of working memory capacity and group as predictors of secondary idea recall

Variable	Lenient method			Strict method		
	*β*	*p*	95% CI	*β*	*p*	95% CI
Intercept	− 0.76	0.86	[− 9.4, 7.9]	3.5	0.19	[− 1.7, 8.6]
WMC	0.22	0.03^*^	[0.04, 0.41]	1.6	0.02^*^	[0.27, 2.9]
Group	2.20	0.70	[− 9.3, 14]	2.4	0.47	[− 4.2, 9]
Interaction	− 0.11	0.39	[− 0.37, 0.15]	− 1.5	0.10	[− 3.3, 0.28]

Formula: “Secondary idea recall” ~ WMC * Group

^*^Statistically significant effect (*p* <.05)

**Fig. 3:**
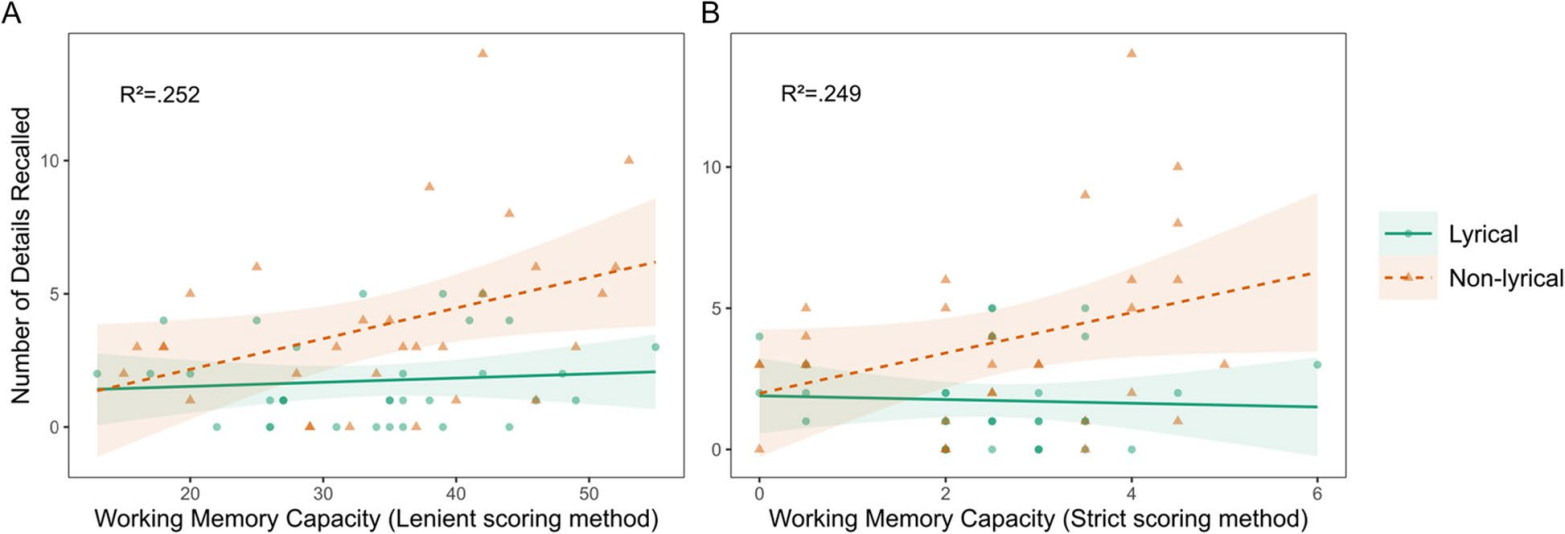
Recall of details predicted by WMC. *Note.* WMC vs number of details recalled, using the lenient method (**A**) and the strict method (**B**) for scoring the reading span test. Colors, shapes and line types differentiate between lyrical and non-lyrical groups. The effect of WMC was statistically significant with both scoring methods and the interaction between WMC and group was statistically significant with the strict scoring method

With respect to recall of details, Table 5 shows a statistically significant effect of WMC with both lenient and strict scoring methods. In addition to that, the strict scoring results showed statistical significance for the interaction between WMC and Group (Fig. 3). On the other hand, there was no statistically significant difference between groups.

**Table 5:** Estimates of working memory capacity and group as predictors of details recall

Variable	Lenient method			Strict method		
	*β*	*p*	95% CI	*β*	*p*	95% CI
Intercept	− 2.30	0.31	[− 4.5, − 0.06]	0.49	0.70	[−0.81, 1.8]
WMC	0.14	0.01^*^	[0.08, 0.18]	0.92	0.01^*^	[0.58, 1.3]
Group	3.70	0.21	[0.8, 6.7]	1.80	0.28	[0.13, 3.5]
Interaction	− 0.13	0.05	[− 0.2, − 0.06]	− 1.10	0.02^*^	[− 1.5, − 0.64]

Formula: “Details recall” ~ WMC * Group

^*^Statistically significant effect (*p* <.05)

As Fig. 3 shows, the higher the WMC, the better recall of details in the non-lyrical group while in the lyrical group there is no visible effect with the lenient method and a slight decrease in recall with the strict method (Fig. 3). Since this statistically significant interaction was observed only with the strict scoring method, whether this is the result of lower statistical power resulting for the non-normal distributions resulting from this method, considering the number of observations, remains unclear.

To better investigate the lack of effects of WMC on the recall of main ideas with the observed effects on the recall of secondary ideas and details, we conducted an exploratory complementary analysis. The analysis examined the correlations between the recall of main ideas, secondary ideas and details across the two groups. If recall scores are positively correlated, then the recall of secondary ideas and details often occurs alongside the recall of main ideas. These correlations, in turn, suggest that recalling secondary ideas and details tends to be more demanding than recalling only main ideas. In other words, these positive correlations would suggest that readers who recall secondary ideas and details usually do so while also recalling main ideas.

**Fig. 4:**
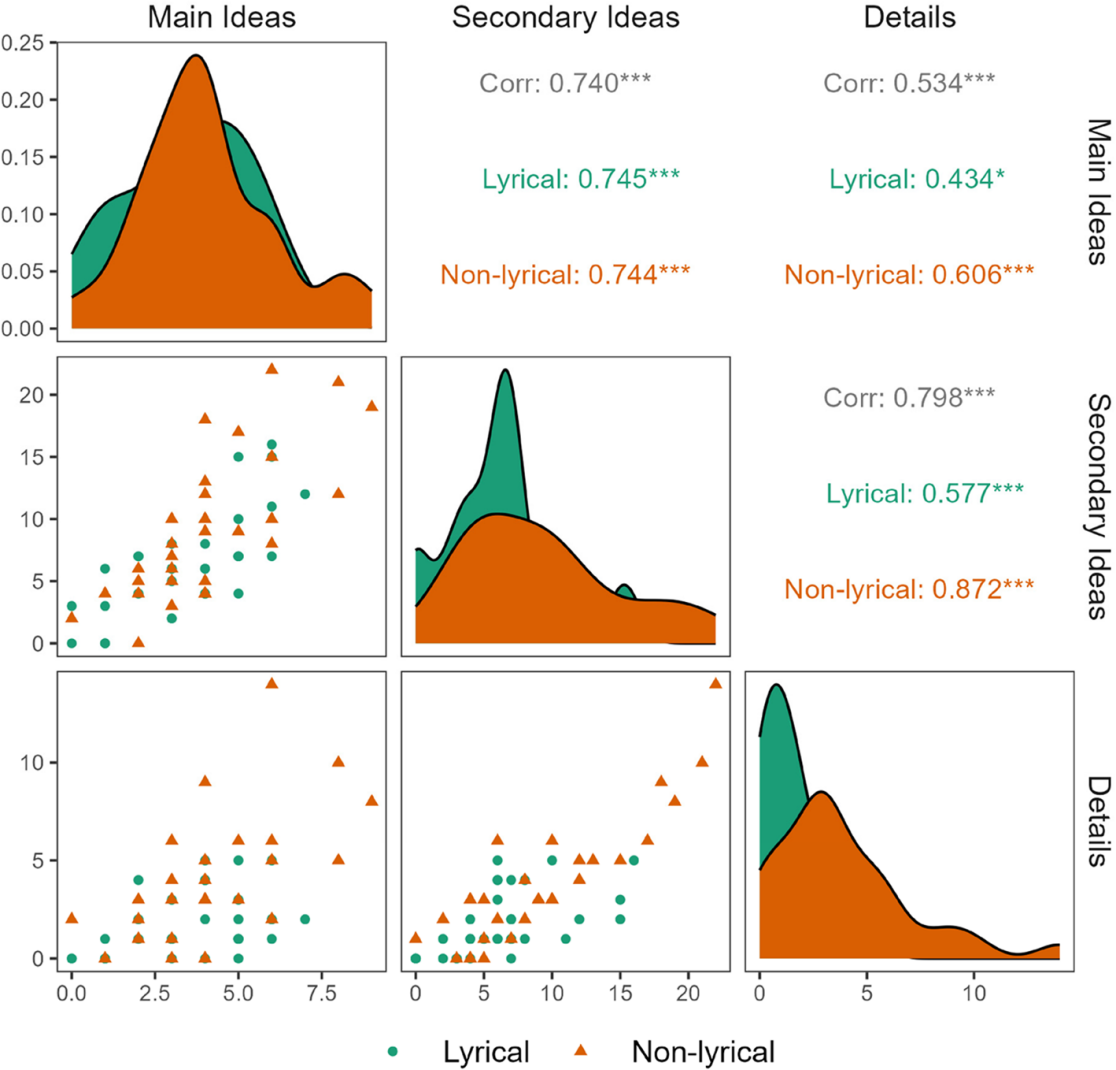
Correlations between idea units recall. *Notes.* Correlations between Main Ideas, Secondary Ideas and Detail recall scores. Colors and shapes differentiate between groups. All correlations are positive and statistically significant. ^*^p <.05, ^***^p <.001

The results of the exploratory complementary analysis are presented in Table 6. Main ideas had the highest probability of being recalled and details had the lowest probability of being recalled in both groups. Figure 4 shows that main ideas, secondary ideas, and detail recalls are positively correlated, that is, participants who recalled more main ideas tended to also recall more secondary ideas and details.

**Table 6:** Means and proportions of the number of idea units recalled

Group	Type	Mean	SE	Proportion
Non-lyrical	Main ideas (MI)	4.07	0.374	0.407
Non-lyrical	Secondary ideas (S)	9.27	1.055	0.309
Non-lyrical	Details (D)	3.83	0.589	0.225
Lyrical	Main ideas (MI)	3.50	0.325	0.350
Lyrical	Secondary ideas (S)	6.21	0.709	0.207
Lyrical	Details (D)	1.74	0.281	0.102

Elaborated by the authors

## Discussion

Considering the results of the main analysis, the data do not support the hypothesis that WMC is a good predictor of main idea recall in a multitasking setting, but it does support the hypotheses that WMC is a good predictor of secondary ideas and details recall. Additionally, the data do not support the hypothesis that non-lyrical music is less distracting than lyrical music. Based on the exploratory complementary analysis, we explain these results with the assumption that the recall of secondary ideas and details, in addition to the recall of main ideas, is more cognitively demanding. In a nutshell, participants with higher WMC recalled more secondary ideas and details than participants with lower WMC in addition to the main ideas that were recalled by all participants, overall. Considering the strict scoring method and specifically the recall of details, higher WMC participants outperformed lower WMC participants in the lyrical group, while no clear effect was observed in the non-lyrical group.

The interpretation that recalling secondary ideas and details is more demanding than recalling main ideas alone holds only if we consider that participants are proficient readers. This implies that they build a mental representation of texts by prioritizing main ideas and assigning less importance to secondary ideas and details. Then, readers would be expected to recall secondary ideas and details in addition to main ideas only if they have sufficient cognitive resources to integrate them after processing the main ideas. Higher WMC readers are expected to have these resources. In other words, readers are unlikely to recall secondary ideas and details without recalling the main ideas, which would invalidate the assumption. As shown in the exploratory complementary analysis, main ideas had the highest proportion of recall (Table 6). Additionally, the recall of main ideas is positively correlated with the recall of secondary ideas and details (Fig. 4), supporting this assumption.

No difference was found between participants from the lyrical vs. non-lyrical groups. The absence of a main effect of Group suggests that participants in the lyrical group were not more distracted than those in the non-lyrical group, as would be expected based on the results by Vasilev et al. (2023). However, the statistically significant interaction observed in the recall of details—when using the strict scoring method – supports the semantic-interference hypothesis (Martin et al., 1988) and the interference-by-process hypothesis (Marsh et al., 2009). This suggests that while all readers integrated main and secondary ideas into their mental representation similarly, regardless of the type of music, higher WMC readers outperformed lower WMC readers in integrating details while listening to lyrical music. Their greater cognitive resources might have allowed them to manage interference from linguistic stimuli from the lyrics more effectively. However, since this interaction was only statistically significant under the strict scoring method, further studies are needed to explore this interpretation in greater depth.

In summary, considering the patterns observed in the complementary analysis, and the results showing no statistically significant effects of WMC nor group in the recall of main ideas but showing statistically significant effects in the recall of secondary ideas and details, the proposed interpretation is that (1) participants focused on main ideas when building their mental representation of texts, thus the absence of effects of WMC in the recall of main ideas can be explained by a lack of high cognitive demand for the addition of main ideas in the mental representation of texts in the population of the study; (2) participants with higher WMC were able to recall more secondary ideas and details in addition to main ideas, which were similarly recalled by all participants, and that is why the effects were only observed predicting secondary ideas and details and not predicting main ideas. However, further studies are necessary to better investigate the proposed interpretation.

A first possible explanation for such findings is connected with participants’ high levels of L2 proficiency and thus, low cognitive demand. Put simply, L2 proficiency alleviates WM capacity, freeing cognitive resources for text processing and recall (Alptekin & Erçetin, 2010). The literature explains that the representation of the global structure of the text—the gist—demands WM resources for chunking information from the text. In order to retrieve the chunked information, both a rich knowledge base and rapid and effortless storage and retrieval operations are needed to avoid overloading WM (van Dijk & Kintsch, 1983). In addition to that, these results seem consistent with previous findings (Oliveira & Tomitch, 2021; Tomitch, 1999), in which more proficient readers (which also tend to be higher-span readers) have been shown to recall more propositions from the text in comparison to less proficient counterparts (which also tend to be lower-span ones) (Tomitch, 1999). In summary, previous studies demonstrated that L2 reading proficiency correlates positively with both WMC and recall scores, with stronger correlations with the latter in comparison with the former, leading to the conclusion that “foreign language reading proficiency is a better predictor of recall than WMC” (Oliveira & Tomitch, 2021, p. 87). It seems, thus, that identifying and/or constructing main ideas from text may be an automatic process for proficient readers, and consequently might not consume all of WM resources (Anderson, 2000). Additionally, scholars tend to agree that skilled reading encompasses the construction or identification of main ideas from texts (Tomitch, 2000, 2003; van den Broek & Kendeou, 2022).

A second possible explanation may relate to the readers’ goal while reading the hypertext used in this experiment. Participants were instructed to read a text and later report the main ideas and all the information they could remember from the text. Thus, participants’ reading goal might have directly influenced their outcomes in the recall task. Similar results were found by Yeari, van den Broek and Oudega (2015), who investigated the effects of reading goals on the recall of central versus peripheral information (analogous to main idea and secondary ideas and details). The authors found that “readers can strategically regulate their overall engagement and selective attention allocation to central and peripheral information in accordance to their reading goals” (Yeari et al., 2015, p. 1092). Thus, goal setting might have moderated reading outcomes.

Finally, our findings were consistent with the notion that main idea summarization may mitigate or counteract a negative effect of multitasking on both integrative processing and integrated understanding of multiple documents (Haverkamp et al., 2024). Presumably, in our study, focusing on main ideas may have led to an effect similar to main idea summarization, which provided a scaffold during multitasking. That means this may have helped readers to identify, select, retain, and organize relevant information that could be drawn on to facilitate integration when reading.

## Conclusion

This study investigated the effects of WMC in L2 digital reading while listening to lyrical and non-lyrical music, considered a multitasking activity. Results showed statistically significant effects of WMC in the recall of secondary ideas and details, but not on the recall of main ideas. A complementary analysis showed that main ideas had a higher probability of being recalled than secondary ideas and details, and that recall of main ideas, secondary ideas, and details are positively correlated. These results led us to the interpretation that WMC may be a good predictor of text ideas recall, more specifically the recall of secondary ideas and details because individuals who recalled secondary ideas and details also recalled main ideas. In other words, while recalling main ideas in a multitasking setting was found to be similar across participants with different WMC, the recall of secondary ideas and details proved to be easier for readers with higher WMC. We assume this was the case because of their ability to cope with the demands of constructing a mental representation of the text that has secondary ideas and details in addition to main ideas. No statistically significant effect of group was observed, suggesting that the difference between lyrical and non-lyrical music did not significantly affect IUs recall in this study. However, a statistically significant interaction between WMC and group was observed in the recall of details with the strict scoring method, which deserves attention in future studies.

These results contribute to a better understanding of the allocation of cognitive resources in L2 digital reading within a multitasking setting. This setting is a common part of many readers’ daily activities, whether as a habit of listening to music while reading or as a consequence of reading in a public space with background music. The results show that proficient readers with higher WMC can manage the demands of this multitasking setting. They can grasp both the main ideas and secondary ideas of a text. However, readers with lower WMC may only be able to comprehend the main ideas.

Consequently, one pedagogical implication of these findings is that lower WMC high-proficient L2 readers, while reading for study purposes, could benefit from avoiding listening to music while reading. This is particularly relevant when the comprehension of main ideas alone may not suffice for desired academic achievement.

Given the correlations between L2 reading proficiency and WMC observed in the literature, a plausible hypothesis emerges. Low proficient readers, especially those with lower WMC, may struggle with the demands of multitasking reading. A pedagogical implication of this hypothesis is that low-proficient L2 readers should also avoid reading while listening to music. This is advisable even if they are accustomed to doing so while reading in their first language. This hypothesis, given its pedagogical implications, warrants further investigation in future studies.

## Data Availability

The datasets used and/or analyzed during the current study are available from the corresponding author on reasonable request. Materials used in this study are available in the Open Science Framework repository, 10.17605/OSF.IO/9F8GH

## Abbreviations

*bpm*: Beats per minute
CI: Confidence interval
D: Details
Hz: Hertz
IQR: Interquartile range
IU: Idea units
*k*: Fleiss’ kappa
L2: Second language
M: Mean
MI: Main idea
*p*: *p*-Value
*R*^2^: *r* Squared or coefficient of determination
S: Secondary ideas
SD: Standard deviation
SE: Standard error
WM: Working memory
WMC: Working memory capacity
*β*: Slope coefficient
